# Shortcutting photorespiration: avenues and challenges toward realizing higher‐yielding photorespiratory bypass crops

**DOI:** 10.1111/nph.70724

**Published:** 2025-11-14

**Authors:** Katherine Meacham‐Hensold, Jooyeon Jeong, Amanda P. Cavanagh, Donald R. Ort

**Affiliations:** ^1^ Carl R. Woese Institute for Genomic Biology University of Illinois Urbana Champaign Urbana IL 61801 USA; ^2^ School of Life Sciences University of Essex Colchester CO4 3SQ UK; ^3^ Department of Crop Sciences University of Illinois Urbana Champaign IL 61801 USA; ^4^ Department of Plant Biology University of Illinois Urbana Champaign Urbana Champaign IL 61801 USA

**Keywords:** food security, photorespiration, photorespiratory bypasses, photosynthesis, thermotolerance

## Abstract

Photorespiration is a major source of photosynthetic inefficiency in C3 crops. In photorespiration, the oxygenation rather than carboxylation of RuBP by Rubisco triggers an energy‐expensive pathway to recycle inhibitory byproducts and recapture lost carbon, ultimately reducing yields. Shortcutting the native pathway through the introduction of photorespiratory bypasses offers a potential route to increase crop yields. In the last decade, several shortcut pathways have been extended from *in vitro* and controlled environment proof‐of‐concept experiments to demonstrated yield increases in replicated field trials. This review summarizes current published bypasses and field trial results, discussing potential and challenges for these alternative pathways to be translated into crop species as a tool for improved food security, and to future‐proof crops for forecast climate change scenarios. Required focus areas to advance theory to reality include a greater understanding of bypass energetics; increased knowledge of the behavior of photorespiratory bypass metabolomic fluxes; exposure of the mechanisms underpinning the thermotolerant properties of some alternative pathways; and the need for collaborative breeder‐style trials in multi‐environment locations to confirm and understand the drivers of photorespiratory bypass crop yield increases at various crop growth stages.

**Table:** 

	Contents	
	Summary	675
I.	Introduction	675
II.	Bypass pathways	677
III.	Bypass energetics	679
IV.	Do photorespiration and bypasses ‘leak’?	681
V.	From proof of concept to proof of technology	684
VI.	Conclusions	686
	Acknowledgements	687
	References	687

## I. Introduction

There are growing incentives to mitigate inefficiencies in photosynthesis as a strategy to increase crop yields as the global population increases (Van Dijk *et al*., 2021), and as the forecasted effects of climate change steadily become reality (Hasegawa *et al*., 2021; FAO, 2024; Bernacchi *et al*., 2025). With the need to grow more food on less land, in stressed conditions, and with limited manufactured inputs, improving the efficiency with which crops utilize the sun's energy to produce calories could have game‐changing benefits for future food production (Long *et al*., 2006; Zhu *et al*., 2010; Simkin *et al*., 2019).

**Fig. 1:**
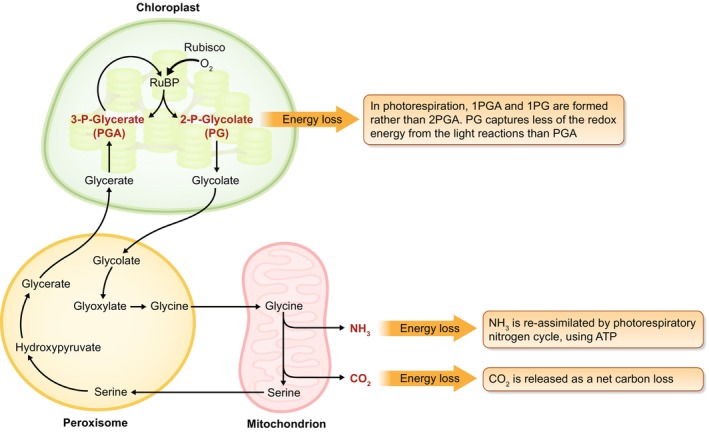
Points of major energy loss in native photorespiration. In photorespiration Rubisco oxygenates RuBP rather than carboxylating it, leading to production of one phosphoglycerate molecule and one phosphoglycolate molecule, a two‐carbon molecule that cannot directly re‐enter the photosynthetic cycle. Energy is lost as two PG molecules produce one PGA and release a previously fixed carbon as CO_2_. Additionally, energy is lost through the release of ammonia (NH_3_) and its reassimilation by the photorespiratory nitrogen cycle. These processes consume ATP and redox power as previously fixed carbon is lost, diverting energy from plant growth and yield. PGA, phosphoglycerate.

Photorespiration is a major source of photosynthetic inefficiency, especially in C3 crops, given the energy cost associated with it (Walker *et al*., 2016; Smith *et al*., 2025). For carbon fixation, ribulose−1,5‐bisphosphate carboxylase‐oxygenase (Rubisco) carboxylates ribulose−1,5‐bisphosphate (RuBP) to produce phosphoglycerate (PGA), a three‐carbon molecule that can re‐enter the photosynthetic cycle, and a portion can be metabolized to produce starch and sugars to contribute to plant growth. In photorespiration, Rubisco oxygenates RuBP rather than carboxylating it, leading to the production of one PGA and one phosphoglycolate (PG), a two‐carbon molecule that cannot directly re‐enter the photosynthetic cycle and can inhibit photosynthetic metabolism (Ogren & Chollet, 1982). The energy cost comes first from combining two PG molecules to produce one PGA, but with the release of a previously photosynthetically fixed carbon as CO_2_ (Campbell & Ogren, 1990; González‐Moro *et al*., 1997; Dellero *et al*., 2016). The second energetic cost of photorespiration involves the release of ammonia and its reassimilation by the photorespiratory nitrogen cycle via glutamine synthetase and glutamine:2‐oxoglutarateaminotransferase. These two native salvage pathways, each involving multiple metabolic steps across four cellular compartments (chloroplast, peroxisome, mitochondria, and cytosol), use ATP and redox equivalents while resulting in the loss of previously fixed carbon, thus redirecting energy from processes related to plant growth and yield (Fig. 1).

Our focus in this review is on alternative pathways that shortcut or bypass the native photorespiratory pathway including the potential and challenges for these alternative pathways to be translated into crop species to increase yield and adapt C3 crops to future climate scenarios. Here, we view native photorespiration in the emerging context of an open cycle that intersects with other aspects of plant cell metabolism (Wingler *et al*., 2000; Bauwe *et al*., 2010; Peterhansel *et al*., 2010; Eisenhut *et al*., 2019; Bauwe, 2023), moving beyond the fixed stoichiometry of classic textbook representations. Many different alternative pathways have been proposed and implemented in plants and for clarity, we have classified the alternative pathways into three types (Table 1): FD, fully decarboxylating pathways; PD, partial decarboxylating pathways; and ND, non‐decarboxylating pathways (i.e. do not release CO_2_).

**Table 1:** Published bypass pathways: categorization, benefits, and drawbacks.

Bypass type	Original designation	Species	Reported benefits	Caveats/drawbacks	Reference
FD1	GMK (Maier) bypass	*Arabidopsis thaliana*	Increased dry weight and higher assimilation rate on both dry weight and Chl basis	Requires precise tuning of enzyme levels; potential H_2_O_2_ toxicity from GO activity	Maier *et al*. (2012)
FD2	GMA bypass	*Oryza sativa*	Increased net photosynthetic rate, biomass, and grain yield without compromising seed‐setting rate; improved source–sink balance	Requires precise inducible promoter design	Xu *et al*. (2023)
FD3	AP3 bypass	*Nicotiana tabacum, Solanum tuberosum, O. sativa*	Increased biomass in field trials; improved photosynthetic efficiency; resilience to elevated temperatures	Requires RNAi suppression of native glycolate transporter PLGG1	South *et al*. (2019); Cavanagh *et al*. (2022); Meacham‐Hensold *et al*. (2024); X. Chen *et al*. (2025)
FD4	GOC bypass	*O. sativa, S. tuberosum*	Enhanced photosynthetic efficiency, biomass, and nitrogen content	Yield variability across seasons and environmental dependency; decreased seed setting rate in rice	Shen *et al*. (2019); Lin *et al*. (2025)
PD0	Partial bypass	*S. tuberosum*	Strongly enhances photosynthesis; elevated sugar and starch levels in leaves led to a 2.3‐fold increase in tuber yield	Requires precise polyprotein design; may need integration with additional approaches to boost sink capacity	Nölke *et al*. (2014)
PD1	GT‐DEF (Kebeish) bypass	*A. thaliana, Camelina sativa, Cucumis sativus*	Increased photosynthesis and biomass production; elevated chloroplastic CO_2_ concentration for RuBisCO refixation	Requires careful construct design to prevent transgene silencing and ensure efficient assembly and stoichiometric expression of multiple subunits of EcGDH	Kebeish *et al*. (2007); Dalal *et al*. (2015); Chen *et al*. (2019)
PD2	GCGT bypass	*O. sativa*	Improved photosynthesis, increased biomass, and higher grain yield	Reduced seed setting rate due to disrupted and imbalanced sugar metabolism in the anthers	Wang *et al*. (2020); Li *et al*. (2024)
PD3	Carvalho bypass	*N. tabacum*	Aimed to reduce ammonia production by bypassing photorespiration in the peroxisome	Lack of HYI expression led to metabolic imbalance, leaf necrosis, increased amino acid concentrations, and reduced sugar levels	Carvalho *et al*. (2011)
ND1	BHAC bypass	*A. thaliana*	Conserves nitrogen; accumulation of C4 metabolites; proof‐of‐principle for a synthetic C4 cycle	Further optimization is needed to achieve the full potential of the BHAC	Roell *et al*. (2021)
ND2/ND3	cBHAC/GCBG bypass	*O. sativa*	Enhanced photosynthetic efficiency and yield; improved nitrogen uptake	Further tuning is needed to maximize bypass flux	G. Chen *et al*. (2025)

## II. Bypass pathways

### 1. CO_2_ ‐releasing bypasses (FD and PD)

CO_2_‐releasing bypasses metabolize the photorespiratory intermediate glycolate and release CO_2_ as a by‐product. The rationale is that relocating CO_2_ release from mitochondria (where it normally occurs in native photorespiration) to the chloroplast could concentrate CO_2_ near Rubisco, potentially boosting refixation. Because glycolate contains two carbons, metabolic engineering can be directed toward complete decarboxylation, releasing both carbons as CO_2_, or partial decarboxylation, which oxidizes one carbon and retains the other.

**Fig. 2:**
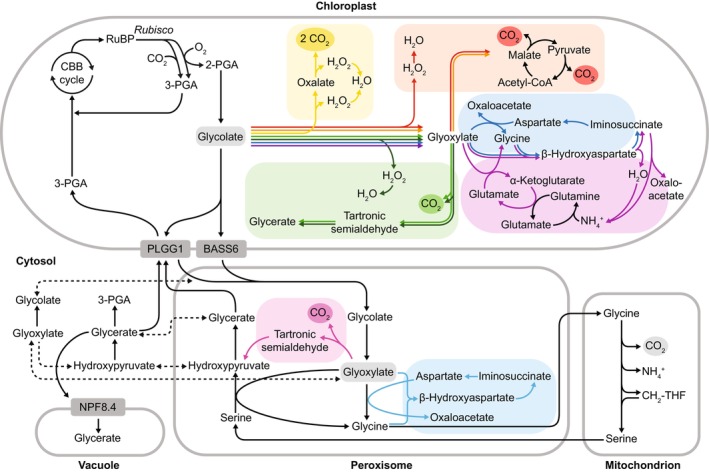
Schematics of different photorespiration bypass pathways. Black arrows denote the native photorespiratory pathway, which spans the chloroplast, peroxisome, mitochondrion, cytosol, and vacuole. Within the chloroplast, Full Decarboxylation (FD) 1 and FD2 bypasses are shown in red (Maier *et al*., 2012; Xu *et al*., 2023); FD3 bypass in orange (South *et al*., 2019); FD4 in yellow (Shen *et al*., 2019); Partial Decarboxylating (PD) 1 bypass in light green (Kebeish *et al*., 2007); PD2 bypass in dark green (Wang *et al*., 2020); Non‐Decarboxylating (ND) 2 and ND3 bypasses in dark blue and purple, respectively (G. Chen *et al*., 2025). Inside the peroxisome, type PD3 is indicated in pink (Carvalho *et al*., 2011) and ND1 bypass is shown in light blue (Roell *et al*., 2021). PLGG1, plastidic glycolate glycerate transporter; BASS6, bile acid Sodium Symporter; PGA, phosphoglycerate.

One prominent example is the full decarboxylation (FD1) bypass introduced by Maier *et al*. (2012) in Arabidopsis, forming malate from glycolate for full oxidation via the transgenic insertion of glycolate oxidase, catalase, and malate synthase into the chloroplast (Fig. 2).

FD1 transgenics showed enhanced photosynthesis and biomass but not O_2_‐dependence of the CO_2_‐compensation point (Γ). Together with an accumulation of TCA cycle intermediates in the transgenics, these results imply that a portion of malate produced may be exported from the chloroplast via the malate valve, and that this carbon redistribution could contribute to the observed growth benefits (Smith *et al*., 2025). Xu *et al*. (2023) then adapted the FD1 bypass in rice by maintaining CmMS but substituting the Maier glycolate‐oxidation module with rice glycolate oxidase (OsGLO1) and ascorbate peroxidase (OsAPX7), calling it a GMA bypass (FD2; Fig. 2; Table 1). By driving glycolate oxidation with an OsGLO1 light‐inducible promoter, the FD2 bypass was associated with higher grain yield without any penalty in seed‐setting rate.

South *et al*. (2019) advanced this concept with the FD3 bypass (Table 1), substituting algal glycolate dehydrogenase in place of glycolate oxidase/catalase for glycolate oxidation. Also, in order to reduce leakage of glycolate out of the chloroplast into the native pathway, expression of the chloroplastic glycolate/glycerate exchange transporter PLGG1 was knocked down (Fig. 2). A lower intercellular CO_2_‐compensation point in the absence of day respiration (C_i_*) confirmed stromal CO_2_ enrichment, and FD3 lines outperformed the wild‐type in glasshouse and field conditions, also contributing thermoprotection under elevated temperature (Cavanagh *et al*., 2022). Utilizing algal glycolate dehydrogenase may have the additional benefit of using plastoquinone as its electron acceptor, thereby capturing redox energy within photosynthetic electron transport that would be lost by glycolate oxidase, which reduces O_2_, forming hydrogen peroxide. The FD3 bypass has been successfully implemented in potato (Meacham‐Hensold *et al*., 2024) and rice (X. Chen *et al*., 2025).

The FD4 pathway (Table 1) is another complete CO_2_‐releasing design demonstrated in rice and potato (Shen *et al*., 2019; Lin *et al*., 2025). Unlike the FD1 bypass, in the FD4 bypass, glycolate is converted to oxalate for full oxidation (Fig. 2). FD4 transgenic lines exhibited a lower chloroplastic CO_2_‐compensation point in the absence of day respiration (Γ*), together with enhanced photosynthesis, biomass, and grain yield. The FD4 bypass also had increased levels of total Chl and total nitrogen relative to the wild type.

These studies validate chloroplast‐targeted complete decarboxylation of glycolate as an effective strategy for boosting productivity. However, a potential trade‐off is that all glycolate carbon is lost as CO_2_, so the net benefit depends on how much of that CO_2_ Rubisco can recapture before escaping the chloroplast. Partial decarboxylating (PD) bypasses (Table 1), which release only a fraction of glycolate carbon, have therefore been explored to achieve a more favorable balance between carbon loss and refixation.

The pioneering study by Kebeish *et al*. (2007) installed a PD1 pathway that originated from the *Escherichia coli* glycolate oxidation pathway. In the PD1 bypass, two molecules of glycolate are converted into one PGA and one CO_2_. PD1 transgenics had a lower Γ* than the wild type, together with higher CO_2_ assimilation rates and greater biomass. The PD1 bypass was later tested in crop species, with Dalal *et al*. (2015) introducing it into camelina and Chen *et al*. (2019) into cucumber. The modified PD2 bypass in rice (Wang *et al*., 2020), which used OsGLO1, also lowered Γ* and enhanced overall performance but showed reduced seed setting, perhaps due to disrupted and imbalanced sugar metabolism in the anthers (Li *et al*., 2024).

These PD transgenic plants showed that even partial decarboxylation can sufficiently raise chloroplast CO_2_ to physiologically relevant levels. Partial bypasses increase chloroplast CO_2_ and save one carbon per glycolate, resulting in a better carbon economy than FD designs. However, partial bypasses still consume ATP and reduce power to re‐fix the single CO_2_ they emit. In principle, a non‐CO_2_‐releasing bypass that recycles glycolate completely without any decarboxylation could achieve even higher energetic efficiency.

### 2. Non‐CO_2_ ‐releasing bypasses (non‐decarboxylating – ND)

Efforts to create ND bypasses were long constrained to *in vitro* systems or cyanobacteria, primarily due to the absence of identified natural enzyme suites to complete such pathways (Shih *et al*., 2014; Ort *et al*., 2015; Trudeau *et al*., 2018). Roell *et al*. (2021) overcame this barrier by transplanting the β‐hydroxyaspartate cycle (BHAC) from marine proteobacteria into Arabidopsis peroxisomes, converting glyoxylate to oxaloacetate in four enzymatic steps without CO_2_ or NH₃ release (ND1; Fig. 2). The oxaloacetate is then presumed to undergo cytosolic export and conversion to a C_3_ intermediate before returning to the photosynthetic cycle, mimicking C_4_ plant metabolism. However, inefficient oxaloacetate reintegration into the photosynthetic cycle constrained PGA regeneration thereby limiting net photosynthesis. Even so, this work provided a proof of concept for a synthetic C_4_ shuttle in C_3_ plants. G. Chen *et al*. (2025) expanded this finding through two strategies. They introduced BHAC directly into chloroplasts (ND2) and, in parallel, designed a new ND3 bypass that coupled BHAC to the chloroplastic nitrogen assimilation (Fig. 2). Both strategies produce chloroplastic oxaloacetates. Metabolite profiling indicated this oxaloacetate could be decarboxylated to PGA, reduced to malate for the TCA cycle, or supply carbon for amino acid synthesis. Collectively, these designs demonstrate the feasibility of engineering non‐CO_2_‐releasing, nitrogen‐conserving bypasses in plants with proof of concept shown via the rice yield increases achieved by G. Chen *et al*. (2025).

### 3. Native detours and engineering considerations

An intriguing finding from Kebeish *et al*. (2007), later supported by Nölke *et al*. (2014) in the PD0 bypass expressed in potato, was that the plant expressing only *E. coli* glycolate dehydrogenase without the other components of PD1 still exhibited improved photosynthetic performance. CO_2_ release from isolated chloroplasts fed radiolabeled glycolate, observed in both wild‐type and transgenic plants, implied the existence of an uncharacterized chloroplastic decarboxylation route (Kisaki & Tolbert, 1969). Plausible scenarios include non‐enzymatic glyoxylate decarboxylation to formate and CO_2_ (Zelitch, 1972) or the plastid‐localized pyruvate dehydrogenase complex accepting glyoxylate and liberating CO_2_ (Blume *et al*., 2013). Although the precise pathway remains elusive, recent metabolic‐flux modeling by von Bismarck *et al*. (2023) again pointed to an alternative chloroplastic glycolate‐processing pathway in the *ggt1* mutant, which lacks the glutamate : glyoxylate aminotransferase 1 enzyme. This alternative pathway produces both CO_2_ and H_2_O_2_, which could help plants rapidly clear PG and elevate intercellular CO_2_ under sudden high photorespiration conditions. Emerging evidence indicates plants can flexibly employ alternative native pathways, such as a cytosolic glyoxylate shunt, a cytosolic glycerate kinase‐mediated route, or glycerate accumulation via a tonoplast glycerate transporter, to accommodate transiently elevated photorespiratory fluxes (Ushijima *et al*., 2017; Lin & Tsay, 2023; Jiang *et al*., 2025). Characterizing these alternative native pathways could aid in designing more effective and flexible synthetic bypasses.

Separately, Carvalho *et al*. (2011) attempted to install a peroxisomal PD3 bypass in tobacco, aiming to avoid mitochondrial photorespiration (Fig. 2). While not enriching chloroplastic CO_2_, this bypass was predicted to reduce the ATP and NADPH costs of ammonium refixation. However, PD3 transgenics exhibited necrosis at ambient CO_2_, elevated free amino acids, and depleted sugars. This phenotype was attributed to compromised nitrogen‐cycle enzyme activities and a consequent ATP : NADPH imbalance. Because photorespiration intimately influences primary metabolism and energy homeostasis, any engineering effort must carefully consider its impact on cellular ATP and NADPH demand, a topic explored in the next section. All reviewed bypass designs are outlined in Table 1 and Fig. 2.

## III. Bypass energetics

Introducing a photorespiratory bypass primarily diverts carbon and nitrogen flow away from the native pathway, reshaping cellular energy demand, internal CO_2_ dynamics, and Rubisco kinetics. These shifts drive the photosynthetic, biomass, and yield outcomes observed in transgenic plants. This section examines the mechanisms underlying each change, reviews their *in vivo* occurrences, and describes experimental methods for detection, including current limitations.

### 1. Energy budget dynamics

Textbook native photorespiration consumes 5 ATP and 3 NADPH per Rubisco oxygenation, including refixation of the 0.5 molecule of CO_2_ released. Building on recent comprehensive reviews of the energy costs shifted away from native photorespiration to various bypasses (Smith *et al*., 2023; Walker *et al*., 2024), we provide energy accounting for new FD2, ND2, and ND3 designs (Table 2).

**Table 2:** Energy demand for various photorespiratory bypass pathways.

Bypass type	Reference	Energy demand for bypass		CO_2_ released		Energy demand for refixing CO_2_		Total energy demand		
		ATP	NADPH	Amount	Location	ATP	NADPH	ATP	NADPH	ATP/NADPH
Native photorespiration		3.5	2	0.5	Mitochondria	1.5	1	5	3	1.67
FD1	Maier *et al*. (2012)	2	−1	2	Chloroplast	6	4	8	3	2.67
FD2	Xu *et al*. (2023)	2	−1	2	Chloroplast	6	4	8	3	2.67
FD3	South *et al*. (2019)	1.36	−1	2	Chloroplast	6	4	7.36	3	2.45
FD4	Shen *et al*. (2019)	2	1	2	Chloroplast	6	4	8	5	1.6
PD1	Kebeish *et al*. (2007)	3	1	0.5	Chloroplast	1.5	1	4.5	2	2.25
PD2	Wang *et al*. (2020)	3	2	0.5	Chloroplast	1.5	1	4.5	3	1.5
PD3	Carvalho *et al*. (2011)	3	2	0.5	Peroxisome	1.5	1	4.5	3	1.5
ND1	Roell *et al*. (2021)	3	2	≤ 0.5	Cytosol	1.5	1	4.5	3	1.5
ND2	G. Chen *et al*. (2025)	2.36	2	≤ 0.5	Cytosol	1.5	1	3.86	3	1.29
ND3	G. Chen *et al*. (2025)	2.86	2	≤ 0.5	Cytosol	1.5	1	4.36	3	1.45

These estimates assume algal glycolate dehydrogenase donates two electrons to plastoquinone (PQ), supporting proton accumulation via the Q cycle, yielding *c*. 0.6 ATP per glycolate. This assumption is supported by the thylakoid membrane association of the enzyme (South *et al*., 2019) and its *in vitro* reduction of artificial electron acceptors (e.g. PMS, DCIP) rather than NAD(P)^+^ (Beezley *et al*., 1976; Aboelmy & Peterhansel, 2014), making PQ a plausible, albeit unconfirmed, electron acceptor in chloroplasts. While bypasses reduce intrinsic ATP and NADPH costs by avoiding several photorespiratory carbon and ammonia refixation steps, their net energetics hinge on the amount of CO_2_ the bypass releases (Table 2) and the amount photosynthesis recaptures. Schemes emitting ≤ 0.5 mol CO_2_ per glycolate retain a calculated energetic advantage, whereas full‐decarboxylation routes significantly increase the net energy cost (Xin *et al*., 2015). Bypasses also alter the ATP : NADPH ratio. Except for the FD4 bypass, the PD1 bypass, and most FD pathways raise this demand above the native photorespiratory value, potentially exacerbating the chloroplast ATP shortfalls. Since linear electron flow produces ATP and NADPH at a 1.29 ratio (von Caemmerer, 2000; Seelert *et al*., 2000), ongoing imbalances can reduce bypass benefits (Strand & Walker, 2023). For the ND2 and ND3 designs, our calculations assume that oxaloacetate re‐enters the photosynthetic cycle. However, *in planta*, oxaloacetate could plausibly be diverted (without decarboxylation) to amino acid synthesis, the malate shuttle/TCA cycle, or other routes (G. Chen *et al*., 2025), potentially allowing the bypass to flexibly adjust its effective ATP : NADPH requirement according to the cell's prevailing energy balance. Incorporating this possible metabolic plasticity into future models might yield more accurate predictions of the net energetic balance and physiological consequences for each bypass.

Recent mathematical models, embedding photorespiratory bypasses in the full metabolic networks, have refined energy budget estimates (Smith *et al*., 2025). This modeling reveals that complete‐decarboxylation bypasses, whose advantages are not apparent in either stoichiometric or kinetic simulations (Xin *et al*., 2015; Basler *et al*., 2016; Smith *et al*., 2023; Walker *et al*., 2024), exhibit benefits under high light and CO_2_‐limited conditions. This finding underscores the importance of incorporating realistic environmental parameters to reconcile discrepancies between *in silico* predictions and observed benefits. Persistent gaps will likely close when models also capture the energy supply and demand dynamics under fluctuating real‐world conditions (Kaiser *et al*., 2015; Moore *et al*., 2021; Fu & Walker, 2023).

### 2. Internal CO_2_ dynamics

Table 2 assumes 0.5 mol CO_2_ released per mol of RuBP oxygenated by Rubisco, based on the accepted biochemistry of the native photorespiratory pathway (Somerville & Ogren, 1981; von Caemmerer, 2000). However, the stoichiometry of CO_2_ release is now understood to vary with environmental factors such as temperature, as evidenced by increased CO_2_ release per oxygenation at elevated temperatures (Hanson & Peterson, 1985, 1986; Walker & Cousins, 2013; Walker *et al*., 2017; Busch, 2020). Walker *et al*. (2017) suggested that this increase may be attributed either to non‐catalytic decarboxylation of photorespiratory intermediates by H_2_O_2_, or to CO_2_ release from catalytic reactions such as starch degradation via the glucose 6‐phosphate shunt or other as‐yet‐unidentified reactions. Experimental evidence now supports the understanding that carbon can exit the photorespiratory pathway as various intermediates, including glycine, serine, and one‐carbon units such as CH_2_‐THF, with CO_2_ release (Harley & Sharkey, 1991; Timm *et al*., 2012; Benstein *et al*., 2013; Busch *et al*., 2018; Busch, 2020; Fu *et al*., 2023). As a result, the higher CO_2_ cost per oxygenation not only lowers glycolate recycling efficiency but also couples with an increased Rubisco oxygenation rate at high temperatures, imposing a dual burden on the photosynthetic carbon balance (Sage *et al*., 2008). In the context of a glycolate‐decarboxylation bypass, an altered CO_2_ stoichiometry where all CO_2_ is released within the chloroplasts could favorably enhance CO_2_ refixation efficiency by mitigating diffusion barriers (e.g. organelle membranes and arrangements) to CO_2_ delivery to Rubisco (Tholen *et al*., 2012; Von Caemmerer, 2013; Ubierna *et al*., 2019). Despite this theoretical advantage, many bypass plants exhibit unintended yet significant alterations in leaf and cellular anatomy that should directly influence CO_2_ diffusion. For example, notable changes in leaf thickness, cell and chloroplast dimensions, and intercellular air spaces have been observed in FD1, FD4, PD1, and PD2 bypasses (Kebeish *et al*., 2007; Maier *et al*., 2012; Dalal *et al*., 2015; Shen *et al*., 2019; Wang *et al*., 2020). Such anatomical modifications may partly be adaptive responses to the elevated stromal CO_2_ induced by bypasses, which can steepen the CO_2_ leakage gradient from the chloroplast to the cytosol (Xin *et al*., 2015). Nevertheless, these structural changes, reported sporadically and varying by species, necessitate systematic and comparative 3D leaf anatomical analysis to establish their prevalence and functional impact.

The CO_2_ compensation point (Γ, Γ*, and C_i_*) serves as an *in vivo* proxy for bypass‐induced alterations in the internal CO_2_ regime. Partial decarboxylation bypasses like PD1 and PD2, which relocate CO_2_ release into chloroplasts, show demonstrably lower Γ* as predicted by biochemical photosynthesis models accounting for CO_2_ diffusion (Kebeish *et al*., 2007; Von Caemmerer, 2013; Wang *et al*., 2020). By contrast, FD designs were predicted to have a higher CO_2_ compensation point due to their doubled CO_2_ release per glycolate (Xin *et al*., 2015). Yet, most FD3 and FD4 engineered plants unexpectedly display lower C_i_* or Γ*, apart from X. Chen *et al*. (2025), where FD1 lines, though anticipated to exhibit a higher O_2_‐dependent Γ than wild‐type, show no difference (Maier *et al*., 2012; Shen *et al*., 2019; South *et al*., 2019; Lin *et al*., 2025). These responses might be rationalized if such bypasses elevate stromal pCO_2_ sufficiently to boost Rubisco carboxylation : oxygenation ratio, thereby counteracting net CO_2_ losses by additional decarboxylation. Chloroplastic CO_2_ enrichment driven by bypass can be quantified via ^12^CO_2_ efflux analysis into ^13^CO_2_ air or gas exchange analysis coupled with carbon isotope discrimination (Busch, 2013, 2020). However, confirming that this enrichment enhances refixation efficiency will require comprehensive, integrated analyses including Rubisco kinetics and targeted metabolite profiling.

### 3. Rubisco dynamics

Altering internal CO_2_ levels by bypass installation could favorably modify Rubisco's *in vivo* carboxylation‐to‐oxygenation ratio. This is particularly significant because the partitioning between carboxylation and oxygenation is directly governed by the CO_2_ and O_2_ concentrations at the enzyme's active site and its intrinsic specificity for CO_2_ over O_2_ (*S*_*c*/*o*_), which typically remains unaltered in bypass transgenics at the same temperature (Jordan & Ogren, 1984; Busch, 2020). Environmental variables such as light intensity, temperature, and water availability influence the internal CO_2_ : O_2_ ratio in plants. Conditions such as high light (via increased photosynthetic CO_2_ draw‐down) or water stress (through stomatal limitation of CO_2_ influx) typically decrease this ratio (Fu & Walker, 2023), while high temperatures further promote Rubisco oxygenation by decreasing CO_2_ solubility in the stroma more than O_2_ and by lowering *S*_*c/o*_ (Brooks & Farquhar, 1985). In this context, CO_2_‐releasing bypasses can elevate chloroplastic CO_2_ concentrations, thereby promoting Rubisco carboxylation. Such effects can be investigated via *in vivo* photosynthetic measurements under varying oxygen conditions, coupled with kinetic modeling of carboxylation and oxygenation rates (Kebeish *et al*., 2007; Busch, 2013; Lin *et al*., 2025). However, auxiliary metabolisms influenced by the CO_2_ : O_2_ ratio also indirectly or directly affect CO_2_ assimilation under certain conditions, necessitating their consideration for comprehensive understanding (Tcherkez & Limami, 2019). In addition to the CO_2_ : O_2_ ratio, Rubisco concentration and its *in vivo* activation state affect performance, though their roles in bypass benefits are less studied (Sage *et al*., 2008). For the FD4 and PD2 bypasses, which increase Chl and chloroplast size, investigating Rubisco content and its activation state could help understand their nitrogen economy (Shen *et al*., 2019; Wang *et al*., 2020).

## IV. Do photorespiration and bypasses ‘leak’?

### 1. Photorespiratory carbon export and reassimilation

Native photorespiration contributes to other metabolic processes beyond the central role in detoxification of PG and regeneration of PGA to the C3 cycle, including redox homeostasis (Ogren, 1984), ROS signaling (Foyer *et al*., 2009), one‐carbon (C1) metabolism (Gashu *et al*., 2025; Eisenhut *et al*., 2019), and nitrogen assimilation into amino acids (Bloom, 2015). Accumulating evidence suggests that photorespiration operates as an open cycle with metabolic export to pathways other than the C3 cycle, which may alter the stoichiometry of carbon released per Rubisco oxygenation and the subsequent energetic budget of photorespiration (Fernie *et al*., 2013; Hodges *et al*., 2016; Busch *et al*., 2018; Fu *et al*., 2023; Walker *et al*., 2024; Jardine *et al*., 2025). Biochemical models of C3 photosynthesis assume the FD of glycine in the conversion to serine (Farquhar *et al*., 1980; von Caemmerer, 2000). However, under higher photorespiratory conditions, such as increased light and O_2_ concentrations, or decreased CO_2_ concentrations, glycine production can outpace downstream metabolism, and pools accumulate (Hitz & Stewart, 1980; Timm *et al*., 2012; Abadie *et al*., 2016; von Bismarck *et al*., 2023). These transient pools store carbon that would otherwise be released through the glycine decarboxylase system, where 2 molecules of glycine release 1 molecule of CO_2_, increasing rates of CO_2_ assimilation above those predicted from steady‐state models alone. In tobacco plants subjected to a temporary increase in oxygen concentration from 2% to 40%, accumulation of the glycine pool contributed to 25% of the observed enhancement in carbon fixation compared to the lower steady‐state baseline (Fu *et al*., 2023). In Arabidopsis, glycine accumulation in the initial stage of a transition from low to high light accounts for *c*. 5–7% of the additional carbon fixed compared to low light conditions (Fu & Walker, 2024). Carbon can also exit the photorespiratory pathway in the form of serine with export rates of *c*. 27–39% measured in tobacco leaves under ambient O_2_ and CO_2_ concentrations using metabolic‐flux analysis (Fu *et al*., 2023). Although this is supported by photosynthetic modeling (Sharkey, 1985; Harley & Sharkey, 1991; Busch *et al*., 2018), serine export is not observed in all species, with a notable lack of export observed in metabolic flux experiments using sunflower (Abadie *et al*., 2016; Abadie & Tcherkez, 2021). As serine is a precursor to nicotine synthesis (Byerrum *et al*., 1956), it may be more readily exported in tobacco compared to sunflower. Photorespiratory serine can also be converted to methylene‐THF, which, upon further reduction to 5‐methyl‐tetrahydrafolate, makes a key contribution to methylation reactions (Hanson & Roje, 2001). In Arabidopsis, 5.8% of assimilated carbon is exported to C1 metabolism via photorespiratory serine under ambient atmospheric conditions (Gashu *et al*., 2025), further highlighting the integration of the photorespiratory pathway with secondary metabolism. The export of these amino acids from photorespiration also imparts a nitrogen cost on photorespiration, which likely explains the link between photorespiration and nitrate assimilation (Rachmilevitch *et al*., 2004).

Although quantified as a net loss, a portion of CO_2_ released during glycine decarboxylation in the mitochondria can diffuse into the chloroplast, where it is reassimilated by Rubisco. To maximize refixation, some C3 species have evolved increased chloroplast coverage of the mesophyll surface surrounding the mitochondria, facilitating the capture and reassimilation of photorespired CO_2_ within the cell (Busch, 2013). Thus, CO_2_ losses of <0.5 per Rubisco oxygenation can occur in native photorespiration, with 29% of photorespired CO_2_ reassimilated in rice, and 26% in wheat at 30°C (Busch, 2013). A key strategy in decarboxylating bypass pathways is the relocation of CO_2_ release from the mitochondria to the chloroplast stroma to enhance Rubisco carboxylation rate relative to oxygenation. However, the benefit of these strategies is inversely related to the plant's existing refixation capacity (Smith *et al*., 2025). Indeed, this natural reassimilation lowers the observed leaf compensation point by 5–10 mmol mol^−1^ CO_2_, depending on chloroplast coverage (Busch, 2013), mimicking the observed impact of photorespiratory bypasses. This suggests that bypasses may provide a lesser benefit in species with enhanced reassimilation capacity (such as rice and wheat) compared to those with relatively poor refixation potential (such as tobacco), but this prediction is not supported by empirical evidence (Table 1). Modeling indicates that fully decarboxylating pathways (Type FD1‐3), which require more energy, do not match the efficiency of native refixation. Instead, these pathways primarily function to recapture CO_2_ that would otherwise be lost and are most effective when operating alongside native photorespiration (Smith *et al*., 2025). This may explain why strategies that aim to enhance flux through bypasses, reducing glycolate efflux from the chloroplast through the downregulation of a glycolate transporter PLGG1, demonstrate a benefit in tobacco, but not in species such as potato or rice (South *et al*., 2019; Cavanagh *et al*., 2022; Xu *et al*., 2023; Meacham‐Hensold *et al*., 2024). Notably, the reassimilation potential of a species is not static and can shift with developmental stage or environmental conditions (Tosens *et al*., 2012; Clarke *et al*., 2021), underscoring the need to account for such variability when engineering bypasses for diverse crop species.

### 2. Implications for bypasses

Although most bypass strategies approach photorespiratory metabolism as a closed metabolic cycle, attempting to divert flux from entering the pathway by metabolizing PG in the chloroplast (Fig. 2), metabolite and carbon export from the native cycle may still be occurring at a relatively high rate. One engineering strategy to mitigate export from photorespiration is to lower flux through the native pathway while installing a synthetic bypass. This strategy has been employed using antisense (South *et al*., 2019; Meacham‐Hensold *et al*., 2024) and CRISPR/Cas9 editing technology (X. Chen *et al*., 2025) to knock down the PLGG1 transporter to prevent PG export from the chloroplast, but this has only been attempted with FD3 pathways. However, carbon and other intermediates can also be diverted from the bypasses and used to synthesize amino acids or other secondary compounds. For example, malate produced from reactions in decarboxylating pathways (Type FD1‐4) could be exported from the chloroplast by the malate valve (see Section I.1). When these additional ‘sinks’ are taken into consideration, stoichiometric metabolic modeling predicts an increased benefit to fully decarboxylating bypasses, as the removal of intermediates limits the subsequent release of CO_2_ from the decarboxylating pathway (Smith *et al*., 2025). Therefore, this leakiness may explain the observed benefits of the decarboxylating bypasses, which have been challenging to reconcile with previous modeling approaches (Xin *et al*., 2015). The canonical role of photorespiration as a recycling pathway that returns carbon back to the C3 cycle is challenged both by the metabolic adaptability of plants containing decarboxylating bypasses that do not return phosphoglycerate to the C3 cycle (Maier *et al*., 2012; Shen *et al*., 2019; South *et al*., 2019), and the high rates of carbon export from the cycle in the form of amino acids (Fu *et al*., 2023). Collectively, this suggests that the next generation bypasses should be designed to consider the integration of photorespiration with other metabolic pathways to deliver the greatest benefit. In particular, the integration between photorespiration and nitrogen assimilation is often highlighted as a risk in efforts to engineer photorespiration (Bloom, 2015; Shi & Bloom, 2021). Environmental suppression of photorespiration (i.e. with elevated CO_2_) is linked to decreased nitrate assimilation and subsequent leaf and grain protein decline (Rachmilevitch *et al*., 2004; Myers *et al*., 2014), suggesting that diverting flux from the native photorespiration pathway may be detrimental to crop quality. To date, no adverse impacts on plant protein content or quality have been reported for bypasses, including those grown in cropping conditions (South *et al*., 2019; Cavanagh *et al*., 2022; Meacham‐Hensold *et al*., 2024). Full nutrition profiles of bypass crops have only been reported for potato expressing the FD3 pathway, where no impact on tuber fiber or micronutrients was observed alongside tuber mass increases (Meacham‐Hensold *et al*., 2024). Future studies should place importance on the nutritional quality of photorespiratory bypass food products, ensuring that any changes in sugar signaling and starch synthesis pathways do not compromise food quality. The GCBG (Type ND2) and cBHAC (Type ND3) pathways were developed to connect synthetic bypass with nitrate assimilation by oxidizing chloroplast glycolate to generate oxa*loacet*ate (G. Chen *et al*., 2025). Rice plants containing either of these bypasses demonstrated increased leaf nitrogen content and grain yield increases relative to WT plants in field trials under low and high nitrogen conditions. Future bypass designs should consider the additional roles that photorespiration plays in plant metabolism.

### 3. The benefits of bypass in high temperatures

**Fig. 3:**
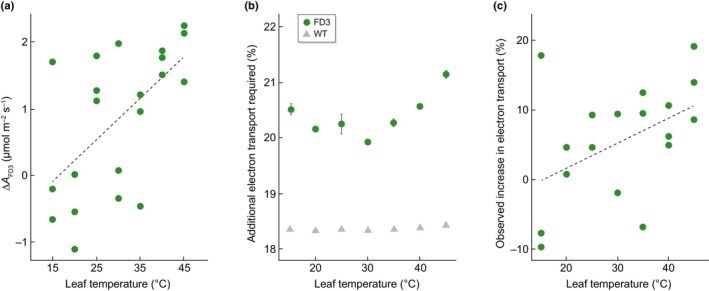
Temperature responses of FD3. Additional carbon assimilation from FD3 (Δ_LD3_) measured as the differential between transgenic and wild‐type (WT) tobacco plants at ambient CO_2_ increases with temperature in FD3 tobacco (redrawn from Cavanagh *et al*., 2022 data) (a). Additional electron transport required to match ATP demand of plants (b). ATP demand and electron transport rates are calculated using assimilation data from Cavanagh *et al*. (2022), using the modeling approaches outlined in Appendix A1. Observed increases in electron transport of PD3 plants relative to WT (c) (redrawn from Cavanagh *et al*., 2022). Error bars represent SEM of *n* = 3 independent transgenic events.

Empirical studies support additional benefits of photorespiratory bypasses with increasing temperature. As temperatures rise, rates of Rubisco oxygenation increase due to decreases in both the relative solubility of CO_2_ relative to O_2_, and Rubisco's specificity for CO_2_ as a substrate relative to O_2_ (Jordan & Ogren, 1984). As a result, oxygenation rates increase, as should flux into the bypass. Kebeish *et al*. (2007) reported that Arabidopsis plants with a PD1 bypass showed consistent growth benefits under both normal and heat stress (35°C), though associated physiological components were not measured. The temperature response of photosynthesis in FD3 tobacco revealed an increased benefit in net carbon assimilation in FD3 transgenic plants relative to unmodified controls as temperatures increase above the thermal optima (Cavanagh *et al*., 2022; Fig. 3a). Over two field trials, FD3 tobacco plants exposed to 5°C of canopy warming demonstrate a thermoprotective effect, reducing the yield penalty by 19% (Cavanagh *et al*., 2022). This is further supported by temperature resilience in field‐grown FD3 transgenic potato plants, which demonstrated increased photosynthetic capacity and tuber biomass in response to natural heatwaves compared to their controls (Meacham‐Hensold *et al*., 2024).

While the mechanisms controlling increased glycolate flux available to the bypasses at elevated temperatures are well understood, the temperature responses of the bypasses themselves are less clear. The presumed primary benefit of the fully decarboxylating pathways such as FD3 is the predicted CO_2_ enhancement effect from the release of additional CO_2_ in the chloroplast stroma. The extra CO_2_ released likely lowers the photorespiratory compensation point in FD3 tobacco and may increase photosynthetic capacity, such as maximal Rubisco carboxylation or electron transport rates (South *et al*., 2019). In FD3 tobacco, changes in the temperature response of photosynthetic capacity do not account for the increases in net assimilation at elevated temperatures, which instead are associated with a lower activation energy of the compensation point (i.e. a less responsive increase with temperature; Cavanagh *et al*., 2022). Empirical data (Cavanagh *et al*., 2022) demonstrate that net carbon gain relative to unmodified controls increases with temperature, suggesting that changes in the stoichiometry of carbon release are occurring in the chloroplast stroma (Fig. 3a). However, due to the stoichiometry of CO_2_ release associated with FD3 pathways (Table 2), assimilation modeled using the Farquhar von Caemmerer Berry (FvCB) model of photosynthesis (Farquhar *et al*., 1980; von Caemmerer, 2000) predicts a loss in net carbon gain as temperatures rise. Therefore, the increased benefit of the FD3 pathway with increasing leaf temperature cannot be explained solely by temperature effects on photosynthesis, as described by the FvCB model.

Modeling the benefit associated with temperature as a function of enhanced CO_2_ fixation ignores other energetic considerations of the pathway (Table 2). For example, as described above, there may also be an energetic benefit if glycolate oxidation via CrGDH reduces PQ and thereby captures a portion of glycolate's redox energy as ATP. Here, we have used the FvCB model to predict the energy demand for additional ATP requirements for FD3 bypass (Fig. 3b). When the stoichiometries of FD3 are accounted for, there is increased electron demand to account for the additional 2.36 mol of ATP the pathway requires (for complete refixation), and the demand strongly increases at leaf temperatures above 30°C (Fig. 3b). Rates of additional electron transport required for WT plants (gray triangles) are consistently *c*. 18% of linear electron flow, aligned with the proportional demand for cyclic electron flow in Arabidopsis plants under similar photorespiratory pressure (Walker *et al*., 2014). In FD3 plants, the putative reduction of PQ by CrGDH could substitute or augment contributions from cyclic electron flow, which would result in observed rates of electron transport through photosystem II that are less than the predicted values. Measured rates of linear electron transport collected over leaf temperatures, indicate that FD3 has higher rates of electron transport at temperatures above 30°C (Cavanagh *et al*., 2022), but the proportional increase relative to WT plants is not high enough to account for the additional demand of ATP (Fig. 3c). Therefore, it is likely that FD3 plants are engaging in some form of additional electron flow mechanism to account for increased ATP production and balance the energy budget. By modeling the additional energy demands of FD3, we show that energy demand is higher in FD3 plants than control plants and this increases with temperature (Fig. 3b). The extra energy demand is partially accounted for by energy savings from the FD3 bypass, particularly at high temperatures over 35°C (Fig. 3c). Observed benefits of FD3 with increasing temperature cannot be explained solely by temperature effects on photosynthesis as described by the FvCB model, which underscores our limited mechanistic insight into bypass operation. More needs to be understood to fully exploit the thermotolerance of FD3 and, likely, other bypass types for yield gains under temperature stress, and more characterization is needed to enable us to model their full benefit at the leaf and crop level (Cavanagh & Matthews, 2025). Full photosynthetic and energetic temperature responses of other bypasses would allow us to better parameterize the FvCB model of photosynthesis to account for the changes in CO_2_ resistances associated with the pathways, and metabolic flux profiling would determine how pathway flux varies with temperature.

## V. From proof of concept to proof of technology

### 1. Photorespiratory bypass field trials

For the carbon‐saving benefits of photorespiratory bypasses to be realized to enhance and increase the stability of food production under future climate scenarios, the bypass function must translate to crop yield increases and resilience under field conditions. Given the many attempts at engineering photorespiratory bypasses *in vitro* and in model crop species, relatively few field studies have been published to date. Those undertaken often come with caveats, contradictions, and challenges.

#### Tobacco

**Fig. 4:**
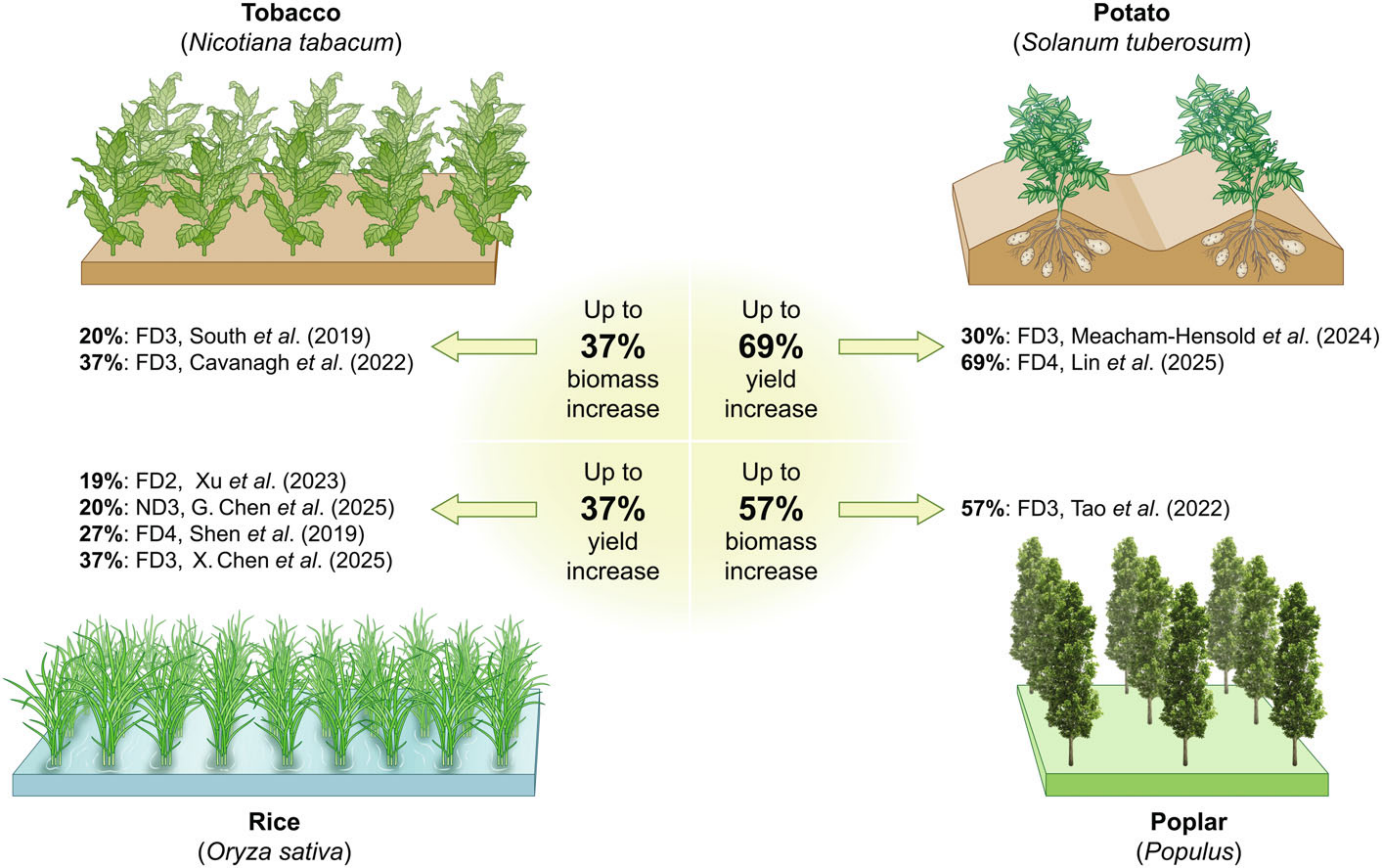
Summarized yield and biomass increases reported in published photorespiratory bypass field trials. Percentage biomass increases are reported for tobacco and poplar, and yield increases for potato and rice.

The work of South *et al*. (2019) tested multiple bypasses in field‐grown tobacco. Three pathways (PD1, FD1, and FD3, Table 1) were measured over two field seasons. While glasshouse testing had shown biomass increases of up to 13%, 18%, and 23% in PD1, FD3, and FD3, respectively, with RNAi knockdown of the PLGG1 transporter, field trials over two seasons only yielded significant increases in biomass compared with controls for FD3 plants. Over two growing seasons, FD3 plants produced up to 24% more biomass compared with untransformed controls (Fig. 4). Gains were supported by increased carboxylation efficiency, increased maximum electron transport rates, reduced CO_2_ compensation points, and shifted photorespiratory metabolite profiles in transgenic plants compared with controls. The work of Cavanagh *et al*. (2022) with FD3 tobacco grown under elevated temperature in field conditions supported the findings of South *et al*. (2019).

#### Potato

With the same FD3 pathway translated into a food crop over two growing seasons, field trials of potato clonally propagated to produce plants heterozygous for the AP3 trait demonstrated tuber biomass increases in FD3 (30%) and FD3 with PLGG1 RNAi (14%). The greatest gains were observed during a season with two natural heatwaves (1–3 d consecutively reaching *c*. 37°C) during early vegetative growth (Meacham‐Hensold *et al*., 2024) (Fig. 4). The benefits in FD3 plants, as in South *et al*. (2019), were associated with increases in maximum carboxylation efficiency (up to 23%), increased maximum rates of photosynthetic electron transport (up to 13%), lower CO_2_ compensation points and shifted accumulation of photorespiratory metabolites compared with controls. However, in a previous growing season, under moderate seasonal temperatures, more modest benefits were seen in FD3 potato, with a 9.5% increase in tuber biomass while FD3 plants with the RNAi module underperformed relative to controls. Observations in field‐grown FD3 tobacco exposed to temperature stress (5°C above ambient control plots) (Cavanagh *et al*., 2022), confirmed the thermotolerant properties of the FD3 bypass at high temperatures (over 35°C), supporting the findings of Meacham‐Hensold *et al*. (2024) in potato. The insertion of a GOC (FD4, Table 1) pathway into clonally propagated heterozygous potatoes by Lin *et al*. (2025) increased photosynthetic rates, leading to tuber biomass increases under both moderate and adverse conditions (low light, frequent storms, and intermittent waterlogging during tuber bulking).

#### Rice

Multiple research groups have translated bypasses into rice for field testing. The introduction of a glycolate oxidase, oxalate oxidase, and catalase bypass (FD4, Table 1) by Shen *et al*. (2019), reported enhanced photosynthetic efficiency, biomass, and nitrogen content, yet did not translate to increased seed yield, likely due to inefficient source–sink metabolism, that is excess photosynthate accumulation in the stem and inefficient conversion of excess CO_2_ to carbohydrate transport to grains (Li *et al*., 2024). To address this bottleneck, Xu *et al*. (2023) expressed the FD2 type GMA bypass (osGLOS1, cmMS, and OsAPX7) (FD2, Table 1) in rice, which in field trials showed increased photosynthetic rates and grain yields without a reduction in seed‐setting rate. Gains were attributed to altered light response kinetics due to the use of the Rubisco small subunit promoter (pRbcS) to drive the expression of OsGLO1 (rice glycolate oxidase), offering moderate increases in photosynthate that did not overwhelm the translation of source to sink. X. Chen *et al*. (2025) also successfully increased rice yield in paddy field trials up to 35% with the insertion of the FD3 pathway (Fig. 4). Similarly, their study aligns with the findings of Meacham‐Hensold *et al*. (2024); in moderate temperature conditions the incorporation of the PLGG1 RNAi knockdown module did not translate to yield increases, possibly due to overwhelming photosynthate accumulation. Another group (G. Chen *et al*., 2025) installed a coupled carbon–nitrogen metabolism bypass (GCBG bypass, Type ND3) into rice, which resulted in enhanced photosynthetic efficiency and grain yield (up to 20%) and improved nitrogen uptake. Further, analysis of metabolites showed that oxaloacetate, produced in the chloroplast from glycolate, aided amino acid and sugar synthesis, likely accounting for the absence of seed‐setting bottlenecks (X. Chen *et al*., 2025).

#### Trees

Inspired by the carbon‐conserving potential of photorespiratory bypasses as a carbon sequestration tool, the climate biotech company, Living Carbon, is attempting to incorporate a version of the South *et al*. (2019) FD3 glycolate oxidation bypass into poplar trees to offer a pathway for mitigating rising global CO_2_ concentrations (Tao *et al*., 2023), with faster plant growth, increased photosynthetic efficiency, and up to 53% increased biomass observed in controlled environment trials over 5 months (Tao *et al*., 2022). Field trials are currently underway, potentially offering climate change mitigation benefits of photorespiratory bypasses beyond increased food production.

### 2. Power, scale, and environmental variation are required to realize bypass crops

The studies outlined here show that photorespiratory bypasses have the potential to increase food crop yields, but published bypass field studies are limited in both number and size. These academic research field trials are the initial stage in finding promising germplasm for further evaluation. While all increases reported are statistically significant, the majority are in a single location with small plot sizes and modest replication (Table 3).

**Table 3:** Location, plot size, and statistical design of published photorespiratory bypass field studies.

Reference	Species	Bypass type	Analysis method	Glycolate	Glyoxolate	Glycine	Serine	Malate	Oxalate	Pyruvate
G. Chen *et al*. (2025)	Rice	ND3	LC‐MS	Reduced	Increased	Reduced	Reduced	Increased	Not reported	Not reported
Shen *et al*. (2019)	Rice	FD4	GC‐MS	Reduced	Reduced 08:00 h, Increased 14:00 h	No change 08:00 h, Reduced 14:00 h	No change	Not reported	Reduced 8:00 h, Increased 14:00 h	Not reported
South *et al*. (2019)	Tobacco	FD3	GC‐MS	Increased	Increased	Reduced	Reduced	Not reported	Not reported	Increased
Meacham‐Hensold *et al*. (2024)	Potato	FD3	GC‐MS	Increased	Not detected	Increased	Increased	Increased	Not reported	Increased

To demonstrate that this technology can enhance global food security, large‐scale breeder trials should be conducted in diverse locations and environments. Tackling such field trials is often beyond the resources of academic institutions and will require public, private, or not‐for‐profit partnerships. This involves complex IP and licensing considerations, negotiations for seed multiplication and testing sites, and multi‐disciplinary collaborative teams. In addition, breeder‐scale field testing in multiple locations is yield‐focused, usually with limited biochemical and physiological analysis. As such, trials of this style will raise more questions about how to fine‐tune constructs for the greatest benefit under varying regimes, likely with the need for incorporation of canopy‐specific and developmental stage targeting promoters, as seen in the work of Jeong *et al*. (2024). Promising germplasm must be refined before product release, requiring close collaboration between inventing researchers and public, private, or not‐for‐profit partners.

Taking FD3 as an example, when the pathway was tested in potato over multiple seasons with different growing season weather conditions, there is evidence to support additional benefits of FD3 after exposure to early vegetative stage heatwaves, but much more work needs to be done to understand temperature thresholds throughout various growth stages. Given the laborious nature of characterizing photosynthesis in the field, characterizing photosynthetic performance with environmental and developmental changes may require sophisticated in‐field phenotyping and the development of a detectable phenotype for characterizing FD3 in field conditions. Seasonal phenotypic characterization must also be coupled with sophisticated meteorological data in order to quantify bypass temperature responses. Such seasonal characterization is necessary to fully exploit bypass thermotolerance and to use targeted promoters to exploit particularly important growth stages and environmental conditions.

### 3. Understanding and utilizing bypass metabolite profiles

Exactly how bypasses function *in planta* remains unclear. Again, using FD3 as an example, when field tested in tobacco (South *et al*., 2019), with and without the RNAi knockdown of the PLGG1 glycolate transporter showed greater benefits than were observed in both food crops potato (Meacham‐Hensold *et al*., 2024) and rice (X. Chen *et al*., 2025). It may be that this RNAi knockdown prevents the native pathway from acting as a safety valve when bypass activity is not great enough to handle all of the glycolate generated in the chloroplast from the FD3 shortcut. CO_2_ compensation points were lowered by the bypass in tobacco and potato, but were increased relative to the control in rice. In particular, the behavior of photorespiratory metabolites can seem contradictory for certain intermediates across all three studies. There is evidence that the FD3 pathway has driven yield increases, but there is a lack of clarity as to the exact mechanisms driving the increases.

In bypass field studies that report photorespiratory metabolites (Table 4), all use gas or liquid chromatograph mass spectrometry to define metabolomics profiles from leaf samples collected at a particular timepoint.

**Table 4:** Photorespiratory metabolomic profiles reported in bypass field studies.

Study	Species	Bypass Type	Location(s)	Plot Size	Statistical Design
South *et al*. (2019)	Tobacco (*Nicotiana tabacum*)	FD3	University of Illinois, Champaign‐Urbana, USA	6 × 6 plants	2016: Randomized single block 2017: complete randomized block with five blocks
Cavanagh *et al*. (2022)	Tobacco (*N. tabacum*)	FD3	University of Illinois, Champaign‐Urbana, USA	12 × 12 plants	Complete block (Experiment 1, *n* = 4; Experiment 2, *n* = 3)
Meacham‐Hensold *et al*. (2024)	Potato (*Solanum tuberosum*)	FD3	University of Illinois, Champaign‐Urbana, USA	10 plants per row plot	Complete randomized block with 6 (2020) and 8 (2022) blocks
Lin *et al*. (2025)	Potato (*S. tuberosum*)	FD4	5 sites in China: Guangzhou, Huan, Gansu, Tianjin, Inner Mongolia	Not Stated	Factorial randomized block design with three replicates.
Shen *et al*. (2019)	Rice (*Oryza sativa*)	FD4	South China Agricultural University, Guangzhou, China	25 m^2^ (5 × 5 m)	Randomized block with four repetitions
Li *et al*. (2024)	Rice (*O. sativa*)	PD2	Not stated	16 m^2^ (4 × 4 m)	Complete randomized block (*n* = not stated)
Xu *et al*. (2023)	Rice (*O. sativa*)	FD2	2 sites: Shanghai and Wuhan, China	16 m^2^ (4 × 4 m)	Complete randomized block with four repetitions
G. Chen *et al*. (2025)	Rice (*O. sativa*)	ND2/ND3	2 sites: CAS Beijing and Zhejiang, China	6 × 6 plants	Complete randomized block with three repetitions
X. Chen *et al*. (2025)	Rice (*O. sativa*)	FD3	Hainan, China	16 m^2^ (4 × 4 m)	Not stated

Using this method, it is only possible to report levels of a particular metabolite at a given timepoint. In this, metabolite levels will be a product of the environmental conditions at the time of leaf sample collections, which explains the contradictory values reported compared with controls across field studies (Table 4). Often, a shift in values for a given metabolite compared with a control can indicate a bypass is operating, but it does not give any mechanistic understanding as to how. Stable isotopic tracers enable metabolic flux analysis to measure reaction rates and can help clarify how introduced bypasses function in field conditions (Allen, 2016; Koley *et al*., 2024), as in the work of Timm *et al*. (2021) in Arabidopsis photorespiratory mutants. This will be crucial moving forward in both understanding bypass responses in different crop species and exploiting bypasses to their full potential in various growing conditions and throughout crop growth cycles.

## VI. Conclusions

Many different alternative photorespiratory pathways have been proposed and implemented in plants, successfully increasing plant biomass and/or yields in controlled environments, offering promise for bypasses as a tool to increase food security. Despite unifying yield or plant biomass increases reported in bypass field trials, this review highlights the contradictions and unknowns that are often seen in the interpretation of the drivers for yield increases. Energetic demands vary greatly among bypass types and are inadequately defined due to the challenges in measuring energetics and tracing the leakiness of the bypass pathways in field trials. Similarly, photorespiratory metabolic flux analysis is required on field‐grown plants at various growth stages to gain greater insight into mechanisms driving yield increases. Bypasses can only provide thermoprotection if they offset the higher energy costs at elevated temperatures, but complex temperature dependencies in field settings make it difficult to optimize seasonal strategies for critical growth periods and temperature stress. For success in achieving higher‐yielding bypass crops, bypass field trials will benefit from seasonal energetic flux characterization, seasonal photorespiratory metabolomic flux analysis, and seasonal characterization of temperature dependencies, all of which are complex to measure and require intensive measurement and analysis campaigns, often requiring collaborative partnerships between research groups.

Most bypass field trials show significant yield gains, but their findings are limited by small size and low statistical power. To move from proof of concept in single‐location research trials to proof that the technology can work to aid global food security, breeder‐style trials need to take place in multiple locations, incorporating a wide range of environmental conditions and at a much greater scale, likely requiring public, private, or not‐for‐profit partnerships. Further, to fully realize yield increases from the transgenically modified photorespiratory bypasses described in this review, scientists must also work with regulators to target in‐country legislative acceptance of genetically modified (GM) food crops and combat societal reluctance toward GM food crops (Lundgren *et al*., 2025).

Photorespiratory bypasses are demonstrating impressive yield increases in controlled environments and single‐location field trials. With collaborative efforts to better understand bypass energetics, metabolomics, temperature responses *in situ*, crop nutritional impacts, expanded size, environmental variation, and statistical power in field trials, and legislative support for GM crop release in target countries, bypasses offer an exciting avenue for increasing crop yield and improving global food security in a changing climate.

## Competing Interests

None declared.

## Disclaimer

The New Phytologist Foundation remains neutral with regard to jurisdictional claims in maps and in any institutional affiliations.

## Modeling theory A1

Assuming complete refixation of photorespired CO_2_ adds additional demand for electrons per Rubisco oxygenation (Table 2). The demand for NADPH and ATP can be estimated by:

(Eqn A1)
$$V_{\text{NADPH}}=2V_{C}+2V_{O}+\left(\alpha \times 1.5V_{O}\right)$$

(Eqn A2)
$$V_{\mathrm{ATP}}=3V_{C}+3.5V_{O}+\left(\alpha \times 1.5V_{O}\right)$$

where *V*_*C*_ is the rate of Rubisco carboxylation, *V*_O_ is the rate of oxygenation, and *α* is the CO_2_ released per oxygenation (which we assume will be refixed here). FD3 also changes the stoichiometry of NADPH/ATP consumption per Rubisco oxygenation (Table 2). If we assume that all glycolate is metabolized through the FD3 pathway (i.e. rates of native photorespiration are negligible), energetic demand can be approximated by:

(Eqn A3)
$$V_{\text{NADP}H_{\mathrm{FD}3}}=2V_{C}+3V_{\mathrm{FD}3}$$

(Eqn A4)
$$V_{{\mathrm{ATP}}_{\mathrm{FD}3}}=3V_{C}+7.36V_{\mathrm{FD}3}$$

where *V*_FD3_ now estimates flux through the pathway. The total amount of electron transport required to support *V*_C_ and *V*_O_ can be calculated from measured rates of carbon assimilation (von Caemmerer, 2000) for native photorespiration (*J*_g_) and the FD3 bypass (*J*_FD3_):

(Eqn A5)
$$J_{g}=\frac{\left(A_{N}+R_{d}\right)\left(4C+5.5\frac{\tau *}{\alpha }\right)}{\left(C-\tau *\right)}$$

(Eqn A6)
$$J_{\mathrm{FD}3}=\frac{\left(A_{N}+R_{d}\right)\left(4C+6\frac{\tau *}{\alpha }\right)}{\left(C-\tau *\right)}$$

Previously measured rates of net assimilation (*A*_N_), respiration (*R*_d_), and photorespiratory compensation point (Γ*) were used for FD3 and WT plants (Cavanagh *et al*., 2022).

Rates of alternative electron flow (FD3 or other) required to balance the NADPH and ATP demand can be calculated following similar approaches to calculate rates of cyclic electron flow (Walker *et al*., 2014), by subtracting the amount of ATP produced by linear electron flow from Eqns A2 and A4, and converted to electron demand assuming 1.5 H+/e− and an ATP synthase requirement of 4.66 H+/ATP, and rearranged to generate an estimate of ATP additional electron transport demand as:

(Eqn A7)
$$\%\text{Electron Flow}=\left(1.51V_{\mathrm{ATP}}-1.95V_{\text{NADPH}}\right)/V_{\text{NADPH}}\times 100$$
